# Risk of endocarditis among patients with coagulase-negative *Staphylococcus* bacteremia

**DOI:** 10.1038/s41598-023-41888-7

**Published:** 2023-09-20

**Authors:** Antonio Ramos-Martínez, Patricia González-Merino, Elena Suanzes-Martín, Marta Murga-de la Fuente, Gabriela Escudero-López, Ane Andrés-Eisenhofer, Esther Expósito-Palomo, Andrea Gutierrez-Villanueva, Itziar Diego-Yagüe, Elena Múñez, Ana Fernandez-Cruz, Jorge Calderón-Parra

**Affiliations:** 1 Internal Medicine Department, Infectious Diseases Unit, Autonomous University of Madrid, Instituto Investigación Sanitaria Puerta de Hierro - Segovia de Arana (IDIPHSA), Hospital Universitario Puerta de Hierro, C/ Maestro Rodrigo 2, 28222 Majadahonda, Spain; 2 Department of Internal Medicine, Hospital Universitario Puerta de Hierro, Majadahonda, Spain; 3 Infectious Diseases Unit, Hospital Universitario Puerta de Hierro, Majadahonda, Spain

**Keywords:** Cardiology, Medical research

## Abstract

Coagulase-negative staphylococci (CoNS) are currently considered typical microorganisms causing infective endocarditis (IE) in patients with prosthetic valves. The objective was to determine variables associated with IE in patients with CoNS bacteremia. We performed an analysis of the clinical characteristics of patients with CoNS bacteremia admitted to a university hospital in Madrid (Spain) from 2021 to December 2022 according to the occurrence of IE. This study is an evaluation of a bacteremia registry. During the study period, 106 patients with CoNS bacteremia were detected. In 85 patients an echocardiogram was performed during hospital admission to rule out IE. Among them, 12 episodes were detected that met IE criteria (14.2%). Of the 6 patients with heart valve prostheses, 5 patients (83.3%) had IE (*p* < 0.001). Patients with IE more frequently had positive blood cultures more than 12 h after the first draw (58.3% versus 13.4%; *p* < 0.001). There was a tendency to associate community-acquired bacteremia and to that all blood culture bottles obtained were positive with an increased risk of IE (*p* = 0.091 and *p* = 0,057, respectively). Attributable mortality to infection was higher in patients with IE relative to all other patients (16.7% vs. 0%; *p* = 0.033). The multivariable analysis included having valve prosthesis and persistent bacteremia for more than 12 h. Both were independently associated with IE: valve prosthesis OR 38.6 (95% CI 5.8–258; *p* < 0.001) and persistent bacteremia OR 2.6 (95% CI 1.1–6.8; *p* = 0.046). In conclusion, a high percentage of cases of CoNS bacteremia may be due to IE. Some of the variables related to a higher risk of IE, such as having a valvular prosthesis or presenting positive blood cultures for more than 12 h, should lead to rule out or confirm the presence of IE by performing echocardiography.

## Introduction

Infective endocarditis (IE) is a feared infection that is associated with high mortality^1,2^. The aging of the population and the increase in invasive diagnostic and therapeutic procedures has led to an increase in the number of healthcare-associated IE cases^1^. One of the main pathogens in these cases are coagulase-negative staphylococci [CoNS) which have a tendency to cause IE on prosthetic heart valves or intracardiac devices^3^.

In recent years, a number of clinical variables have been recognized that are associated with the risk of IE in patients with bacteremia caused by *Staphylococcus aureus*, *Enterococcus faecalis* and non β-hemolytic streptococci^4–9^. The analysis of these variables has allowed the preparation of scores that help to identify patients with indication for echocardiography.

Although many cases with positive blood cultures for CoNS are associated with mild catheter infections or even interpreted as mere contamination, at present these microorganisms are considered typical pathogens of IE on prosthetic valves^2,10^. IE due these microorganisms is characterized by heart failure in a large number of patients, great valvular destruction, periprosthetic abscesses and high mortality^11^. Unfortunately, no studies have been conducted to identify variables associated with IE in patients with CoNS bacteremia.

The objective of this study was to determine variables associated with IE in patients admitted with CoNS bacteremia. We aimed to develop a score able to predict the risk of IE in these patients. This would allow us to indicate tests to confirm or rule out IE, mainly echocardiography.

## Methods

From January 2021 to December 2022, 930 consecutive patients with bacteremia were prospectively included in the database in a 600-bed tertiary care hospital in Madrid, Spain. The registry included demographic, clinical, microbiological, echocardiographic, clinical management, and prognostic sections. Mortality at 30 days after the onset of bacteremia was considered. The cohort registry was approved by the local ethics committees. The Institutional Review Board of the Hospital Universitario Puerta de Hierro (Madrid, Spain) approved the study protocol (Number PI_2923). The informed consent was waived by the “Comité de Ética de Investigación del Hospital Puerta de Hierro”. All methods were performed in accordance with the relevant guidelines and regulations.

### Definitions

Chronic renal failure was defined as a baseline glomerular filtration rate < 60 ml/min per 1.73 m^2^^12^. Patients with positive blood culture interpreted as contaminant were excluded from the registry. In the setting of coagulase negative identification, positive blood cultures were considered true bacteriemia if: (1) The patient had symptoms related to possible infection (temperature ≥ 37.2 °C, tachypnea, tachycardia, hypotension), and (2) Positive blood culture bottle from more than 1 different extraction of the same coagulase negative species with the same antibiogram. Otherwise, the positive blood culture was considered a contaminant. Bacteremia of community origin and healthcare-associated bacteremia were accounted for as previously published^13^. Persistent bacteremia was defined as persistent positive blood cultures 12 h after the first blood culture was obtained in patients on antibiotic treatment. Only episodes of endocarditis that met the criteria for definite endocarditis were considered^14^. Attention was paid to identify patients with surgical indications and, within this group, those who were not operated on. In order to consider that a patient had a surgical indication, the European guidelines currently in practice were taken into account^2^ In cases where a patient presented several episodes of CoNS bacteremia, only the first episode was considered.

### Statistical analysis

Categorical variables are expressed as absolute numbers and percentages. Quantitative variables are expressed as median and interquartile range (IQR). Categorical variables were compared using the χ^2^ test or Fisher's test when necessary. Quantitative variables were compared using the Mann–Whitney U test. In the comparison of IE risk factors, those variables with *p* < 0.05 in the univariate analysis and that were considered clinically significant were included in a multivariable logistic regression model.

In order to construct the predictive score, we performed a second logistic regression model including variables associated with IE (*p* < 0.10) and clinically relevant in the univariate model. Then, we assigned points to each variable proportionally to their β-coefficient in the model. The score calibration was assessed using the Hosmer–Lemeshow goodness-of-fit test and its discrimination though the area under the receiver operating characteristic (ROC) curve. Sensitivity, specificity and predictive values are also provided. The best cut-off point was selected based on J-point. Bilateral p-values below 0.05 were considered statistically significant. All statistical analyses were performed using SPSS version 25 software (SPSS Inc, IBM, Chicago, Illinois, United States.

## Results

During the study period, out of a total of 981 episodes of true bacteremia, 106 patients were caused by CoNS (10.8%), with 85 episodes having an echocardiogram performed during hospital admission. Of these, In 12 patients the bacteremia was due to IE (14.2%). Transesophageal echocardiogram was performed in all patients with IE versus 23 patients (31.5%) without IE (*p* < 0.001).

### Risk factors of infective endocarditis in patients with CoNS bacteremia

Table 1 shows demographic, comorbidities and clinical characteristics of patients with and without IE. Age was higher in patients with IE, but without being statistically significant (*p* = 0.088). Regarding comorbidity, there were more patients with systemic autoimmune diseases in patients with IE (33.3% versus 7.4%, *p* = 0.025). Of the 6 patients with cardiac valve prosthesis, IE was demonstrated in 5 patients (83.3%) (*p* < 0.001). There was a tendency for community-acquired bacteremia to be associated with an increased risk of IE. Specifically, 41.7% of patients with IE had community-acquired bacteremia versus 19.2% in patients who did not develop IE (*p* = 0.091). Patients with IE more frequently had persistent bacteremia (58.3% versus 13.4%; *p* < 0.006). There also was a tendency that all blood culture bottles obtained were positive in patients with IE (75 vs. 41.1%, *p* = 0.057). Both overall mortality (33.3% versus 6.8%; *p* = 0.025) and mortality attributable to bacteremia (16.7% versus 0%; *p* = 0.024) were higher in patients with IE. The two variables included in the multivariable analysis regarding the risk of developing IE were having valve prosthesis and persistent bacteremia for more than 12 h. Both were independently associated with IE: valve prosthesis odds ratio (OR) 38.7 [95% confident interval (CI) 5.8–258, *p* < 0.001] and persistent bacteremia OR 2.7 (95% CI 1.1–6.7, *p* = 0.045).

**Table 1:** Clinical variables in patients with CoNS bacteremia according to whether or not it was associated with IE.

	IE (n = 12)	No IE (n = 73)	*p*
Demographic and comorbidities			
Age (years)	75 (64–80)	66 (55–75)	0.088
Gender (female)	41.7% (5/12)	42.5% (31/73)	1.000
Simple Charlson Index	3 (1–4)	2 (1–4)	0.360
Age-adjusted Charlson Index	6 (4–8)	4 (3–7)	0.086
Arterial hypertension	66.7% (8/12)	47.9% (35/73)	0.367
Diabetes mellitus	50.0% (6/12)	26.0% (19/73)	0.093
COPD	8.3% (1/12)	12.3% (9/73)	1.000
Chronic renal failure	16.7% (2/12)	24.7% (18/73)	0.720
Hemodialysis	0	6.8% (6/73)	1.000
Solid organ malignancy	33.3% (4/12)	16.2% (11/68)	0.224
Hematologic malignancy	0	27.9% (19/68)	0.060
Autoimmune disease	33.3% (4/12)	7.4% (5/68)	0.025
Chronic liver disease	0	5.5% (4/73)	0.620
Dementia	16.7% (2/12)	13.7% (10/73)	1.000
Recent SARS-CoV2 infection	8.3% (1/12)	6.8% (5/73)	1.000
Previous cardiac conditions			
Chronic heart failure	41.7% (5/12)	21.9% (16/73)	0.167
Native valve diseases	25.0% (3/12)	11.0% (8/73)	0.350
Prosthetic valve	41.7% (5/12)	1.4% (1/73)	< 0.001
CIED	8.3% (1/12)	6.8% (5/73)	1.000
Bacteremia clinical presentation			
Community-acquired	41.7% (5/12)	19.2% (14/73)	0.091
More than 7 days of symptoms	25.0% (3/12)	9.6% (7/73)	0.146
Fever	100% (12/12)	88.2% (60/68)	0.462
Severe sepsis	25.0% (3/12)	19.1% (13/68)	0.698
Septic shock	0	4.4% (3/68)	1.000
SOFA (points)	2 (1–3)	2 (0–4)	0.977
Pitt index (points)	1 (0–2)	0 (0–2)	0.218
Prolonged (> 12 h) bacteremia*	58.3% (7/12)	13.4% (9/68)	0.003
*Polymicrobial bacteremia*	0	9.6% (7/73)	0.459
*Staphylococcus epidermidis*	75.0% (9/12)	58.9% (43/73)	0.353
*Staphylococcus hominis*	9,1% (1/12)	27.4% (20/73)	0.156
*Staphylococcus haemolyticus*	9,1% (1/12)	9.6% (7/73)	0.890
*Staphylococcus capitis*	9,1% (1/12)	2.7% (2/73)	0.331
*Staphylococcus schleiferi*	0	1.4% (1/73)	0.683
Meticilin-resistant CoNS	50.0% (6/12)	76.1% (51/67)	0.083
All blood culture bottles positive	75.0% (9/12)	41.1% (30/73)	0.057
Transesophageal echocardiogram performed**	83.3% (10/12)	30.1% (22/73)	< 0.001
Outcome			
Effective initial antibiotics	50.0% (6/12)	60.3% (44/73)	0.507
Source control recommended and not performed***	25.0% (3/12)	4.1% (3/73)	0.034
30-day all-cause mortality	33.3% (4/12)	6.8% (5/73)	0.025
Bacteremia-related 30-day mortality	16.7% (2/12)	0	0.024

CoNs: Coagulase-negative staphylococci. IE: infective endocarditis. COPD: chronic obstructive pulmonary disease. CIED: Cardiac implantable electronic device. SOFA: sequential organ failure assessment (SOFA). *Control blood cultures were not available in 5 patients without infective endocarditis vs 0 patients with infective endocarditis, *p* = 0.614. **Vegetation present in 83.3% (10/12) of transesophageal echocardiogram and in 41.7% of transthoracic echocardiogram (5/12). Two patients with negative transesophageal echocardiogram were diagnosed based on PET-TAC**. ***Seven patients had surgical indications, of which 5 patients underwent surgery. The two patients who did not undergo surgery died during hospitalization. Qualitative variables are expressed as percentage (numerator/denominator) and compared by means of Chi-squared test (or Fisher exact test whenever necessary). Quantitative variables expressed as median (IQR) are compared by means of Mann–Whitney’s U.

### Developing of predictive risk score

To calculate a risk score, the following variables were considered: valve prosthesis, community-acquired bacteremia, bacterial growth in all bottles obtained, persistent bacteremia for more than 12 h after the initial blood culture. We assigned points to each variable according to the logistic regression result: valve prosthesis (4 points), community-acquired bacteremia (2 points), growth in all blood culture bottles obtained (2 points), positive blood cultures separated by more than 12 h (1 point). The score scores were based on beta coefficients obtained using a one-step multivariate logistic regression model whose variables are shown in Table 2.

**Table 2:** Predictive score of IE risk among patients with true CoNS bacteremia.

Variable	Beta-coefficient	Points
Valve prothesis	3.71	4
Community-acquired bacteremia	2.03	2
Bacterial growth in all obtained bottles	2.07	2
Persistent bacteremia for more than 12 h	0.75	1
Total	–	9

Beta-coefficients were obtained by including all variables in a single-step multivariate logistic regression model. Variables entered on step 1: Heart valve prosthesis, community-acquired bacteremia, CoNS isolated in all bottles, persistent bacteremia (> 12 h apart). IE: infective endocarditis. CoNs: Coagulase-negative staphylococci.

**Figure 1:**
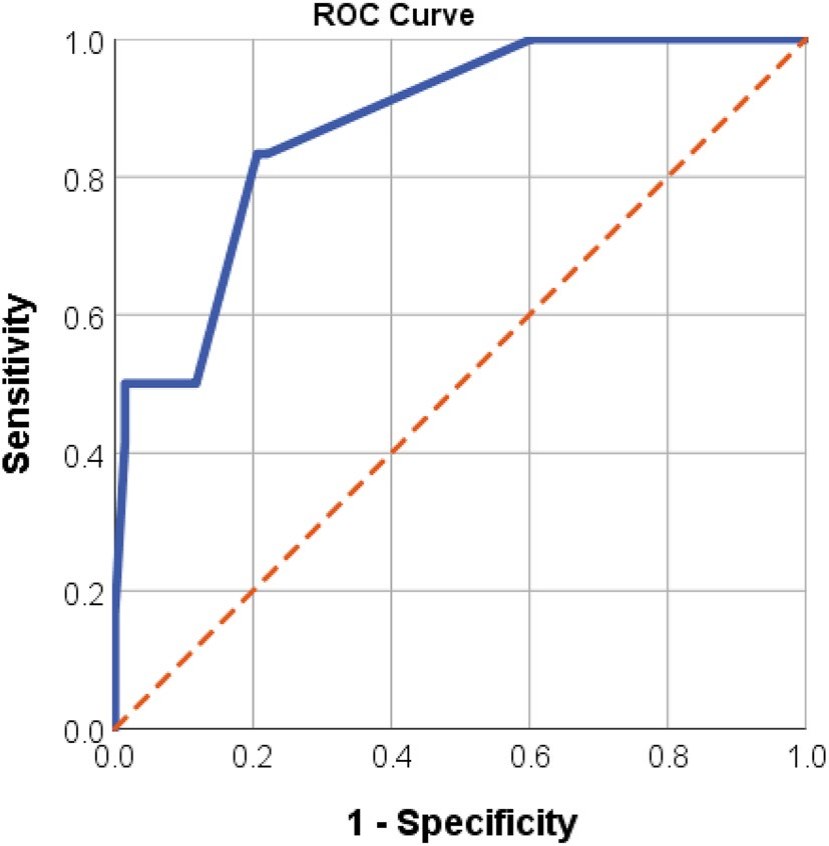
Receiver operating characteristic (ROC) curve considering a specific scores to risk factors for IE in patients with CoNS bacteremia. The score assigned points to each variable as follows: valve prosthesis (4 points), community-acquired bacteremia (2 points), growth in all blood culture bottles obtained (2 points), positive blood cultures separated by more than 12 h (1 point)). AUC: 0.875 (CI 95%: 0.770- 0.974, *p* < 0.001). IE: Infective endocarditis. CoNs: Coagulase-negative staphylococci.

The score was well calibrated (Hosmer–Lemeshow test *p* = 0.217). The score showed a very good discrimination, with an area under the ROC curve of 0.87 (95% confident interval 0.77–0.97, *p* < 0.001, Fig. 1). The risk of IE was clearly progressive with the number of points obtained in the score: 0% for 0–1 point (0/27), 7.1% for 2 points (2/28), 30.7% for 3–4 points (4/13) 44.4% for 5–7 points 5–7 points (4/9) and 100% for > / = 7 points (2/2). Five patients had a missing value in one or more of the variables of the score. Table 3 shows the sensitivity, specificity, positive and negative predictive values of different cut-off points. The J-point was 3 points. In a population similar to ours, a punctuation in the score lower than 2 points would have a negative predictive value of 100% (thus, being the diagnosis of IE highly unlikely), and a punctuation equal or higher than 2 points would have a positive predictive value of 21.4%. Moreover, a patient with a punctuation equal or higher than 4 points would have a very high IE risk (positive predictive value of 41.1%).

**Table 3:** Sensitivity, specificity, positive predictive value and negative predictive value of different cut-off points of the score in patients with an echocardiogram performed.

Cut-off point	Sensitivity (%)	Specificity (%)	Positive predictive value (%)	Negative predictive value (%)
≥ 2 points	100	35.3	20.2	100
≥ 3 points	100	39.7	21.4	100
≥ 4 points	83.3	80.4	41.1	96.7
≥ 5 points	50.0	98.5	84.5	92.2
≥ 6 points	16.7	100	100	87.9

Positive and negative predictive values are calculated using a population prevalence equal to the prevalence found in our registry (14.2%).

## Discussion

The main findings of the study were the high percentage of patients with CoNS bacteremia who had infective endocarditis (11%) and the marked association of this infectious complication with having a heart valve prosthesis.

CoNS are one of the more commonly microorganism found in both primary and secondary bacteremia. They are also a very prevalent as etiologic agent for catheter-associated bacteremia^15^. Unfortunately, CoNS frequently contaminate blood cultures because they are normal constituents of skin flora^15^. However, our results suggest that IE should be considered in patients with risk factors for this complication. Within CoNS, *S. epidermidis* was the most frequent cause of IE, as observed in other studies^3,16^. A slight tendency for *S. epidermidis* to cause IE and *S. hominis* to cause bacteremia without endocarditis was observed (Table 1). However, this trend was far from statistical significance, perhaps because of the small number of patients studied. In contrast to the good prognosis of other infections originating from CoNS^17^, IE on both native and prosthetic valves can be associated with valve destruction, heart failure, periprosthetic abscesses and high mortality, especially in patients with surgical indications who do not undergo surgery^13,16,18^.

The incidence of IE in patients with CoNS bacteremia in the present study was higher than that described in other series^19,20^, but lower than that described for *S. aureus* bacteremia^4,21,22^. The higher incidence in our patients could be related to the high percentage of patients with valve prostheses compared to other studies^19,20^.

### Risk factors of Infective Endocarditis in patients with CoNS bacteremia

In recent years, studies have been carried out to identify risk factors for endocarditis in patients with bacteremia caused by other microorganisms typical of this complication. These risk factors include predisposing cardiac pathology, the presence of implantable intracardiac devices or valve prostheses, community acquisition of bacteremia, clinical presentation, and the characteristics of the blood cultures obtained^4–8^. Several differences have been observed in the association of the clinical variables above mentioned with the risk of IE appearance in patients with bacteremia due to *S. aureus*, *Enterococcus faecalis* and *Streptococcus* spp. This may be related to the characteristics of the patients and the different biological behavior of these bacteria. In the case of bacteremia due to CoNS, we have not found previous studies that have studied risk factors in IE patients.

In the univariate analysis we found that age exhibited tendency to be higher in patients with IE, which may be related to higher comorbidity and lower resistance to infection^20^. Likewise, autoimmune diseases, heart valve prosthesis, and prolonged bacteremia of more than 12 h were significantly associated with the risk of IE. The community origin of bacteremia and positivity of all blood culture bottles obtained showed a tendency to be associated with IE but did not reach statistical significance. The incidence of IE in patients with autoimmune diseases is an aspect not well studied, although there are studies that suggest an increased risk of IE in these patients related to immunosuppression^23^ .

The most important result of the present study was the high risk of IE in patients with valve prostheses. Staphylococci, as a whole, are characterized by their capacity for adherence, invasion, persistence in tissues and evasion of immunity^15^. Although the virulence of CoNS is lower than that of *S. aureus*, CoNS have a high capacity to adhere to artificial surfaces through the production of various adhesins and the formation of biofilms^15,24,25^. On the other hand, and considering that IE produces a continuous bacteremia, it was to be expected that an association between the risk of endocarditis and persistence of bacteremia would have been evidenced^19,26,27^.

### Developing of predictive risk score

Another important result of our study is the developing of a predictive risk score. Using this score, we were able to classify patients in low and high risk of IE (punctuation equal or greater than 2 points. By means of the use of this new tool, echocardiography studies, especially transesophageal echocardiography, could be directed to those with high risk. On the opposite, these studies could be withheld in those patients with very low risk (score punctuation lower than 2 points). This approach is similar to that is being studied for bloodstream infections caused by other bacteria that usually cause IE, including *S. aureus*, *E. faecalis*, and streptococci^28–30^. Nevertheless, such score has not been developed before for CoNS bacteremia and could be a clinical useful tool.

### Limitations

This study has several important limitations. Firstly, it is a retrospective study in a single hospital with a limited number of patients. These characteristics may limit the generalizability of the results. Secondly, we should point out that both the number of blood culture bottles and the time at which they were obtained during the follow-up of the patients showed some differences between them, which may have influenced the validity of the results obtained. In addition, the small number of cases of endocarditis has also reduced the number of variables to be considered in the multivariable analysis, which may have prevented the identification of independent variables associated with the risk of IE. Finally, t should also be noted that the characteristics of the study only allowed us to obtain the score for calculating the risk of endocarditis based on the result of the univariate analysis. Nevertheless, we consider that this work could be a starting point to try to establish more solidly the risk factors for endocarditis in patients with CoNS bacteremia.

## Conclusions

In conclusion, our study demonstrates that a high percentage of cases of CoNS bacteremia may be due to IE. The fact that the patient presents some of the variables related to a higher risk of IE, such as having a prosthetic valve, should prompt us to rule out or confirm the presence of IE by performing imaging tests such as echocardiography. Future studies should confirm the variables most strongly related to the risk of IE in patients with CoNS bacteremia.

## Data Availability

The datasets used and/or analyzed during the current study are available from the corresponding author on reasonable request.
